# CD73 is associated with poor prognosis in HNSCC

**DOI:** 10.18632/oncotarget.11435

**Published:** 2016-08-20

**Authors:** Zhen-Hu Ren, Cheng-Zhong Lin, Wei Cao, Rong Yang, Wei Lu, Zhe-Qi Liu, Yi-Ming Chen, Xi Yang, Zhen Tian, Li-Zhen Wang, Jiang Li, Xu Wang, Wan-Tao Chen, Tong Ji, Chen-Ping Zhang

**Affiliations:** ^1^ Department of Oral Maxillofacial-Head and Neck Oncology, Ninth People's Hospital, Shanghai Jiao Tong University School of Medicine, Shanghai 200011, China; ^2^ Shanghai Research Institute of Stomatology and Shanghai Key Laboratory of Stomatology, Shanghai 200011, China; ^3^ Department of Oral Pathology, Ninth People's Hospital, Shanghai Jiao Tong University School of Medicine, Shanghai 200011, China

**Keywords:** CD73, adenosine receptor, EGFR, EMT, head and neck squamous cell carcinoma

## Abstract

CD73 is a cell surface immunosuppressive enzyme involved in tumor progression and metastasis. While patients whose cancer cells express elevated CD73 are typically associated with an unfavorable outcome, the clinical impact of CD73 expression in patients with Head and neck squamous cell carcinoma (HNSCC) remains unclear. In the present study, we investigated the prognostic significance of CD73 in HNSCC using gene and protein expression analyses. Our results demonstrate that high levels of CD73 are significantly associated with reduced overall survival in patients with HNSCC. We also investigated the functional role of CD73 *in vitro* and demonstrated that CD73 promotes HNSCC migration and invasion through adenosine A3R stimulation and the activation of EGF/EGFR signaling. Moreover, *in vivo* xenograft studies demonstrated that CD73 promotes tumorigenesis. In conclusion, our study highlights a role for CD73 as a poor prognostic marker of patient survival and also as a candidate therapeutic target in HNSCCs.

## INTRODUCTION

Head and neck squamous cell carcinoma (HNSCC) is the sixth most common cancer in the world, accounting for nearly 3% of all cancers. HNSCC is the most common and lethal histopathological type of head and neck cancer with a 5-year survival rate of about 50% [1, 2]. Each year, 500,000 patients will receive a new diagnosis of HNSCC [3]. Despite significant research advances in our understanding of the prevention, diagnostics, and treatment strategies for this disease, the survival rate of patients with HNSCC has shown minimal improvements [4–10]. The mortality rate of HNSCC remains high due to resistance to therapy, driving local recurrences and distant metastases. Thus the development of novel therapies and improved understanding of the mechanisms underlying HNSCC invasion and metastasis represent issues of critical, clinical significance.

CD73 is a 70-kD, glycosyl-phosphatidylinositol (GPI) anchored cell surface enzyme encoded by *NT5E*, also known as 5′-nucleotidase (5′-NT) or ecto-5′-nucleotidase (ecto-5′-NT). CD73 was originally described as a lymphocyte differentiation antigen, shown to catalyze the dephosphorylation of extracellular adenosine monophosphate (AMP) to adenosine [11, 12]. In addition to its enzymatic function, CD73 is also an adhesive and signal molecule mediating cancer invasive and metastatic properties by regulating cell interaction with the extracellular matrix (ECM) [13, 14]. Indeed, the enzymatic and non-enzymatic functions of CD73 are not completely independent of each other and both have been noted to be involved in cancer-associated processes.

CD73 is overexpressed in many types of cancer, associated with unfavorable clinical outcomes [15–18]. While increasing evidence has suggested that CD73 is a key regulatory molecule in cancer development, the clinical importance of CD73 in HNSCC remains unclear. Our previous studies have suggested that high levels of CD73 are associated with a worse prognosis in patients with HNSCC. However, we still know very little about the role of CD73 in the pathobiology of HNSCC.

The objective of this study was thus to assess the prognostic significance of CD73 in human HNSCC, and to further explore the role of CD73 in cancer cell invasion and metastasis.

## RESULTS

### CD73 expression is associated with lymph node metastasis and poor prognosis

**Figure 1:**
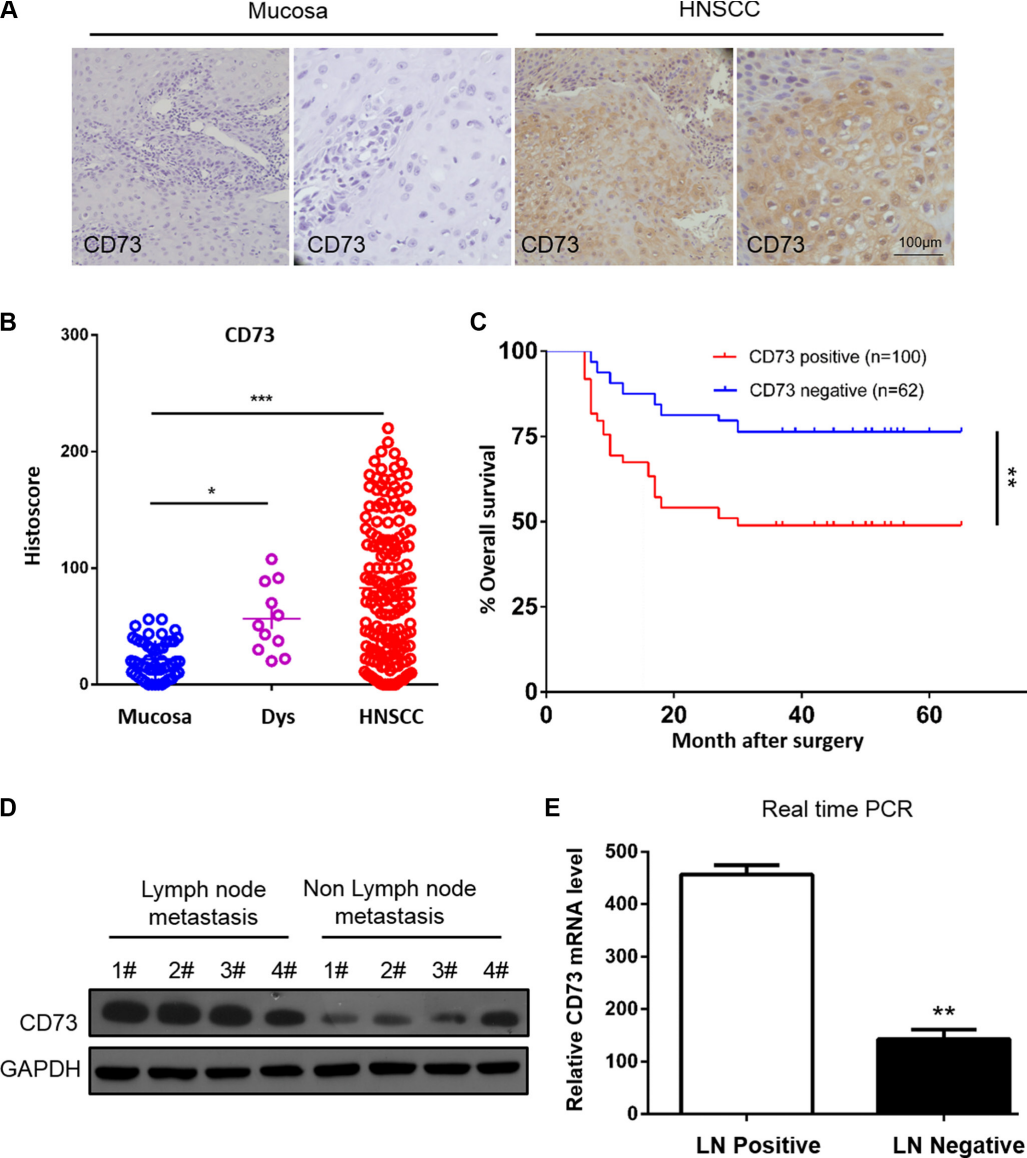
CD73 expression is upregulated and associated with poor prognosis in Head and Neck Squamous Cell Carcinoma (HNSCC) (**A**) Representative immunohistochemical staining (IHC) of CD73 in head and neck squamous cell carcinoma tissue (left) as compared to normal oral mucosa (right) (*Scale bars* = 100 μm); (**B**) Quantification of histoscores of CD73 expression in normal oral mucosa (*n* = 51), epithelial dysplasia (*n* =11) and HNSCC (*n* = 162), *P < 0.05, ***P < 0.001; (**C**) Patients with high CD73 expression have a poor prognosis as compared to patients with low expression (*P* = 0.002, *n* = 162), Significance of differences in survival between patient groups was estimated by log-rank test; (**D**) Western blot analysis of the protein expression of CD73 in patients with/without Lymph node. GAPDH was used as loading control; (**E**) Relative CD73 mRNA expression was detected by RT-PCR in patients with versus without Lymph node metastasis. The data were presented as the means ± SEM. One-way ANOVA with post-Dunnett analysis was performed using GraphPad Prism 5. *P < 0.05, **P < 0.01 versus the control group. (*n* = 3).

To assess protein expression of CD73 in human HNSCC, we performed immunohistochemical staining of CD73 in human HNSCC tissue including normal oral mucosa (*n* = 51), epithelial dysplasia (*n* = 11) and HNSCC (*n* = 162). We found that CD73 is mostly located in the cell membrane and cytoplasm of HNSCC cells (Figure 1A). Quantification analysis revealed that CD73 expression in epithelial dysplasia and HNSCC is strongly positive as compared to the normal oral mucosa (*P* < 0.05 and *P* < 0.001, Figure 1B). Using follow-up data from 162 HNSCC patients, we plotted Kaplan–Meier overall survival curves and analyzed whether CD73 expression affected overall survival (OS). Positive staining was observed in 61.7% (100 of 162) of HNSCC samples. Kaplan–Meier survival analysis demonstrated that positive expression of CD73 was significantly associated with a poor 5-year OS (*P* = 0.002; Figure 1C). Multivariate Cox regression analyses revealed that CD73 expression is an independent prognostic factor for poor OS (*P* = 0.003; Table 1). Moreover, the relationship between CD73 expression and clinicopathological characteristics were evaluated, highlighting a direct association between CD73 expression and lymph node metastasis (*P* < 0.0001; Table 2). To further explore whether CD73 is associated with HNSCC progression we compared CD73 expression in different grades, T categories and N categories of HNSCC (Figure S1A, S1B and S1C). While no difference was observed between different Grades or T categories, we did identify a statistically significant difference between CD73 expression in specimens with lymph node metastasis (N+) and versus those where lymph node metastasis was not observed (N−). Expression of CD73 protein levels and mRNA levels were examined in eight fresh tumor samples (four N+ and four N−) by Western blot and real time PCR. Both CD73 protein levels and mRNA levels in N+ patients were higher than those observed in N− patients (Figure 1D and Figure 1E).

**Table 1:** Cox proportional hazards regression models in estimating cancer development

Variables	Overall survival		*P*
	HR	95%	
**Univariate analysis**			
CD73 expression			
Positive VS negative	**2.472**	**1.367–4.469**	**0.003**
Gender			
Male VS female	1.017	0.512–2.020	0.961
Age			
< 60 VS ≥ 60	1.008	0.983–1.033	0.539
Smoking			
Yes VS no	0.790	0.378–1.650	0.530
Drinking			
Yes VS no	0.910	0.422–1.962	0.811
Tumor stage			
3–4 VS 1–2	**1.355**	**1.025–1.791**	**0.033**
Lymph node metastasis			
+ VS −	**1.676**	**1.230–2.282**	**0.001**
Clinical stage			
3–4 VS 1–2	**1.433**	**1.034–1.986**	**0.031**
Histological type			
Poor VS well-moderate	1.312	0.986–1.744	0.062
**Multivariate analysis**			
CD73 expression	**2.370**	**1.306–4.466**	**0.005**
Lymph node metastasis	**1.765**	**1.203–2.348**	**0.002**

CI, confidence interval; HR, Hazard ratio.

**Table 2:** Association between the patient's clinicopathological characteristics and CD73 expression in 162 HNSCC patients

Clinicopathological features	No.	CD73	expression	*X*^2^	*P*
		Negative (%)	Positive (%)		
Gender				0.981	0.277
Male	100	35 (35.0%)	65 (65.0%)		
Female	62	27 (43.5%)	35 (56.5%)		
Age, years				0.768	0.238
< 60	87	36 (41.4%)	51 (58.6%)		
≥ 60	75	26 (34.7%)	49 (65.3%)		
Smoking				0.282	0.360
Yes	59	21 (35.6%)	38 (64.4%)		
No	103	41 (39.8%)	62 (60.2%)		
Drinking				0.872	0.226
Yes	46	15 (32.6%)	31 (67.4%)		
No	116	47 (40.5%)	69 (59.5%)		
Tumor stage				3.453	0.056
3–4	33	8 (24.2%)	25 (75.8%)		
1–2	129	54 (41.9%)	75 (58.1%)		
Clinical stage				4.753	**0.021**
3–4	67	19 (28.4%)	48 (71.6%)		
1–2	95	43 (45.3%)	52 (54.7%)		
Lymph node metastasis				4.175	**0.000**
+	52	10 (19.2%)	42 (80.8%)		
−	110	52 (47.3%)	58 (52.7%)		
Histological type				0.136	0.460
Poor	14	6 (42.9%)	8 (57.1%)		
Well- Moderate	148	56 (37.8%)	92 (62.2%)		

### The effect of CD73 on the invasion, migration and EMT process in HNSCC cell

**Figure 2:**
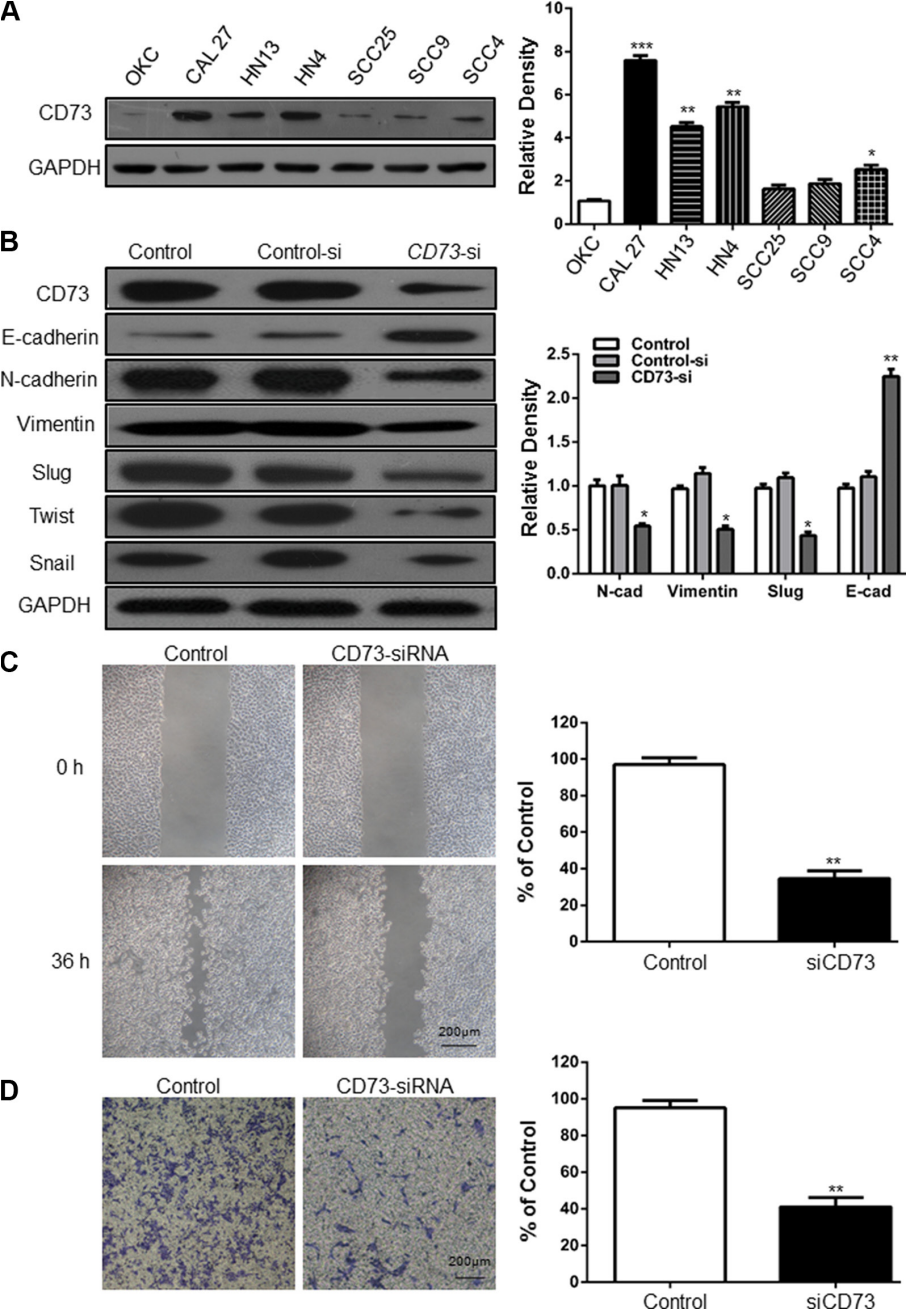
Knockdown of CD73 decreases migration and invasion in HNSCC cell lines (**A**) CD73 expression was detected in one normal oral epithelial cells and six HNSCC cell lines. The relative intensity of CD73, in each cell line, divided by the intensity of GAPDH; (**B**) E-cadherin, N-cadherin, Vimentin, Twist, Snail and Slug levels in. CAL27 cells were evaluated following siRNA –mediated suppression of CD73. GAPDH was the internal standard for protein loading. The values are presented as the means ± SEM. One-way ANOVA with post-Dunnett analysis was performed using GraphPad Prism5. *P < 0.05, **P < 0.01 versus the control group.(*n* = 3); (**C**) Knockdown of CD73 resulted in suppressed cell mobility of CAL27 cell line, and quantification of cell numbers with ImageJ “cell counter” module shows the statistical significance of the difference (Mean ± SEM; **P < 0.01, student *t*-test with GraphPad Prism5.0), (*Scale bars* = 200 μm); (**D**) Cell migration abilities of CAL27 were impaired following knocking down of CD73, compared with those of control group, and quantification of cell numbers with Image J “cell counter” module (Mean ± SEM; **P < 0.01, student *t*-test with GraphPad Prism5.0, (*Scale bars* = 200 μm).

To determine whether the CD73 is involved in invasion and/or migration of HNSCC, we performed *in vitro* assays using HNSCC cell lines. As shown in Figure 2A, CD73 expression was upregulated at the protein level in HNSCC cell lines (CAL27, HN13, HN4, SCC25, SCC9, SCC4) relative to normal keratinocytes (OKC). CAL27 and HN4 cell lines had the highest expression of CD73 and were used for subsequent *in vitro* studies. To evaluate the impact of CD73 on invasion and migration of HNSCC cells, siRNAs targeting CD73 were designed (Figure 2B). Wound healing and Boyden chamber invasion assay demonstrated that CD73 knockdown notably decreased the cell mobility of CAL27 cell line, leading to a significant difference in the number of migrating cells between the control group and the siRNA treatment group, respectively (*P* < 0.01) (Figure 2C). Similarly, knockdown of CD73 also significantly decreased the number of invasive cells as compared with those observed in the control group (*P* < 0.01) (Figure 2D).

To better understand the mechanism by which CD73 modulates metastasis of HNSCC, we explored the effect of CD73 on Epithelial-Mesenchymal Transition (EMT), via the presence/absence of epithelial marker E-cadherin, N-cadherin, Vimentin, Twist, Snail and EMT transcriptional factor Slug. Weston Blot analysis demonstrated that knockdown of CD73 suppressed the protein expression of N-cadherin, Twist, Snail and Slug, partially suppressed Vimentin, but increased the expression of E-cadherin in CAL27 cell line (Figure 2B). These findings suggest that knockdown of CD73 may inhibit EMT progression.

### CD73 promotes invasion and metastasis of HNSCC through adenosine A_3_ and A_2A_ receptor stimulation

**Figure 3:**
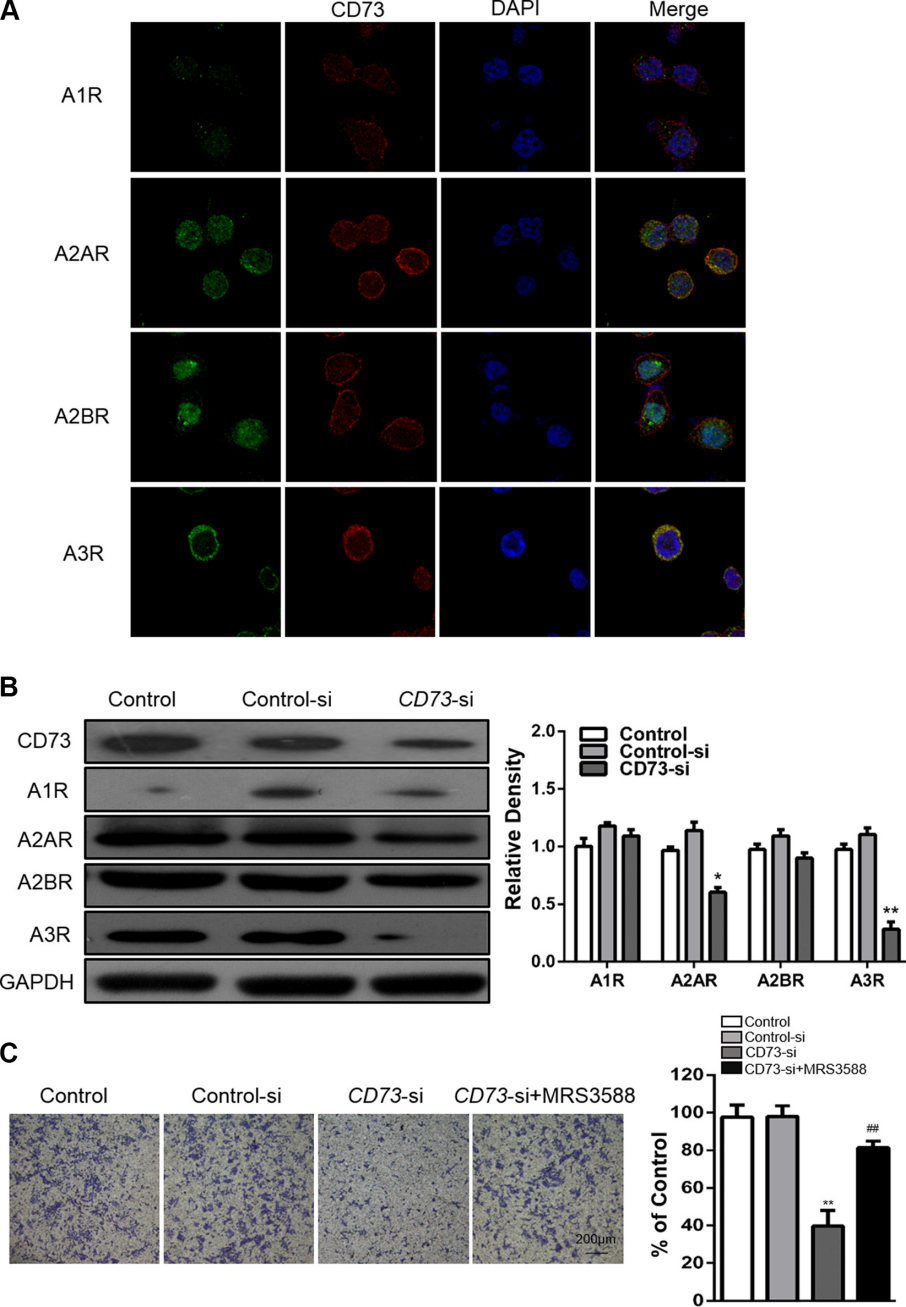
CD73 promotes invasion and metastasis of HNSCC through adenosine A3 receptor stimulation (**A**) Confocal immunofluorescence microscopy of CD73 and A_1_R, A_2A_R, A_2B_R, A_3_R co-expression in CAL27 cells; (**B**) CAL27 cells were treated with siRNA for CD73, then the A_1_R, A_2A_R, A_2B_R and A_3_R levels were determined. GAPDH was the internal standard for protein loading. The values are presented as the means ± SEM. One-way ANOVA with post-Dunnett analysis was performed using GraphPad Prism5. *P < 0.05, **P < 0.01 versus the control group. (*n* = 3); (**C**) Transwell assay demonstrated that CAL27 cell invasion was impaired following knocking down of CD73 compared with those of control group. These findings could be reversed using MRS3588, an agonist of A_3_R, quantification of cell numbers with Image J “cell counter” module (Mean ± SEM; **P < 0.01, versus control-si group, ^##^*P* < 0.01, versus the CD73-si group, student *t*-test with GraphPad Prism5.0), (*Scale bars* = 200 μm).

Surface bound CD73 converts AMP to adenosine. Adenosine, acting through G-protein coupled receptors (i.e. adenosine A_1_, A_2A_, A_2B_, and A_3_ receptors), has been known to promote tumor growth, migration and invasion. However, the effect of CD73′s enzyme activity on the migration and invasion of HNSCC cancer cells has not been investigated. Our results demonstrate that CD73 is co-expressed with A_3_R and A_2A_R (Figure 3A). Western blot results confirm that when expression of CD73 was suppressed, the expressions of A_3_R and A_2A_R were also reduced (Figure 3B). To address the contributions of A_3_R to the migration and invasion-promoting effects of CD73, we treated the CAL27-siRNA cells with MRS3588 – a specific A_3_R agonist. Upon treatment with MRS3588, cell migration and invasion significantly increased (Figure 3C and Figure S2A), while treatment with other adenosine receptor agonists did not alter these properties (Figure S2B–S2G). Taken together, these data demonstrated that CD73 promotes HNSCC migration and invasion via the adenosine A_3_R stimulation.

**Figure 4:**
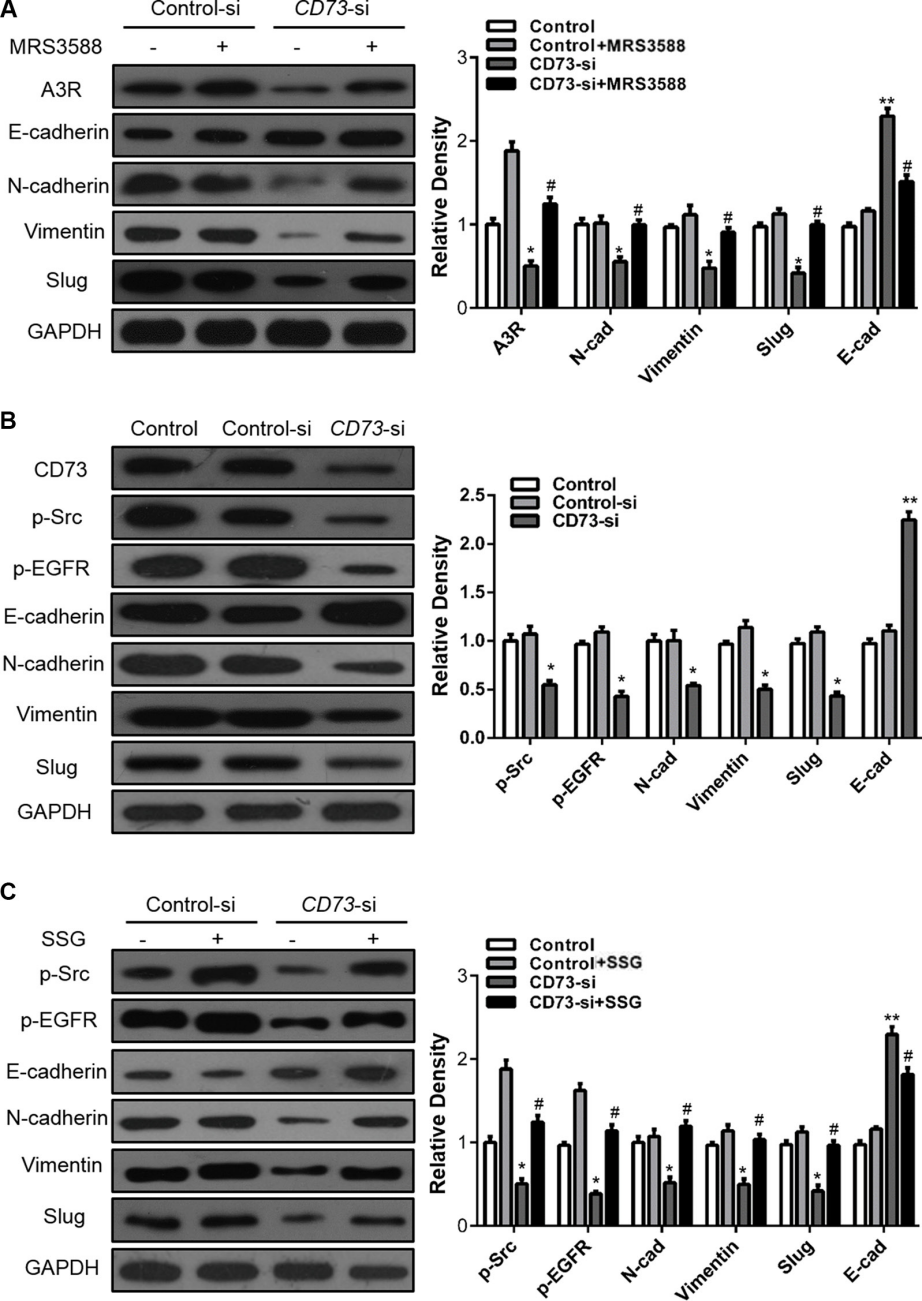
EGFR and adenosine signaling pathways are involved in CD73-dependent EMT in HNSCC cells (**A**) CAL27 cells were treated with siRNA targeting CD73 or control siRNA followed by incubation with MRS3588 for another 36 h. E-cadherin, N-cadherin, Vimentin and Slug levels were evaluated. GAPDH was the internal standard for protein loading. One-way ANOVA with post-Dunnett analysis was performed using GraphPad Prism5.*P < 0.05, versus the si-control group, ^#^*P* < 0.05, versus the CD73-si group. (*n* = 3); (**B**) CAL27 cells were treated with siRNA for CD73, then the p-Src, p-EGFR, E-cadherin, N-cadherin, Vimentin and Slug levels were determined. GAPDH was the internal standard for protein loading. The values are presented as the means ± SEM. One-way ANOVA with post-Dunnett analysis was performed using GraphPad Prism5. *P < 0.05, **P < 0.01 versus the control group.(*n* = 3); (**C**) CAL27 cells were treated with siRNA against CD73 or control siRNA followed by incubation with SSG for another 24 h, then the p-Src, p-EGFR, E-cadherin, N-cadherin, Vimentin and Slug levels were determined. GAPDH was the internal standard for protein loading. One-way ANOVA with post-Dunnett analysis was performed using GraphPad Prism5. *P < 0.05, versus the siRNA-control group, ^#^*P* < 0.05, versus the CD73-si group. (*n* = 3).

To better understand the mechanism by which A_3_R contributes to the changes in migration and invasion mediated by CD73, we treated both the CAL27-control and CAL27-siRNA cells with MRS3588, and detected changes in E-cadherin, N-cadherin, Vimentin and EMT transcriptional factor Slug. Knockdown of CD73 suppressed expression of N-cadherin and Slug, partially suppress Vimentin expression, and resulted in increased expression of E-cadherin in the CAL27 cell line. Notably, MRS3588 could reduce the changes induced by knockdown of CD73 (Figure 4A).

### CD73 promotes invasion and metastasis of HNSCC through EGFR signaling pathway

siRNA-mediated suppression of CD73 in cancer cells led to downregulation of p-EGFR and p-Src (Figure 4B), while SSG (a Src activator) treatment led to a further decrease in p-EGFR expression. This finding demonstrates that CD73 regulates EGFR phosphorylation via p-Src (Figure 4C). When CD73 expression was suppressed by siRNA, inhibition of p-EGFR, p-AKT, p-FAK and p-ERK was observed. Meanwhile, the expressions of EGFR, AKT, FAK and ERK were not affected (Figure S3A). To validate our findings, we performed Western blot analysis to detect changes in EGFR signaling pathway at 5 min, 15 min, 30 min, 45 min, 1 h, 4 h, 12 h, 24 h following EGR treatment. Our results demonstrated that EGFR signaling pathway was activated within 5 min of EGF treatment. Moreover, p-EGFR was rapidly degraded in the CD73-siRNA group, with a degradation speed in the CD73-siRNA group that was faster than that observed in the control group (Figure S3B). Grey value curves were generated using Image-J software (Figure S3C) and the area under curve of control group was greater than that of CD73-siRNA group, suggesting that CD73 promoted downstream signaling of EGF/EGFR.

### Targeting CD73 blocks tumor growth, invasion and metastasis of HNSCC *in vivo*

**Figure 5:**
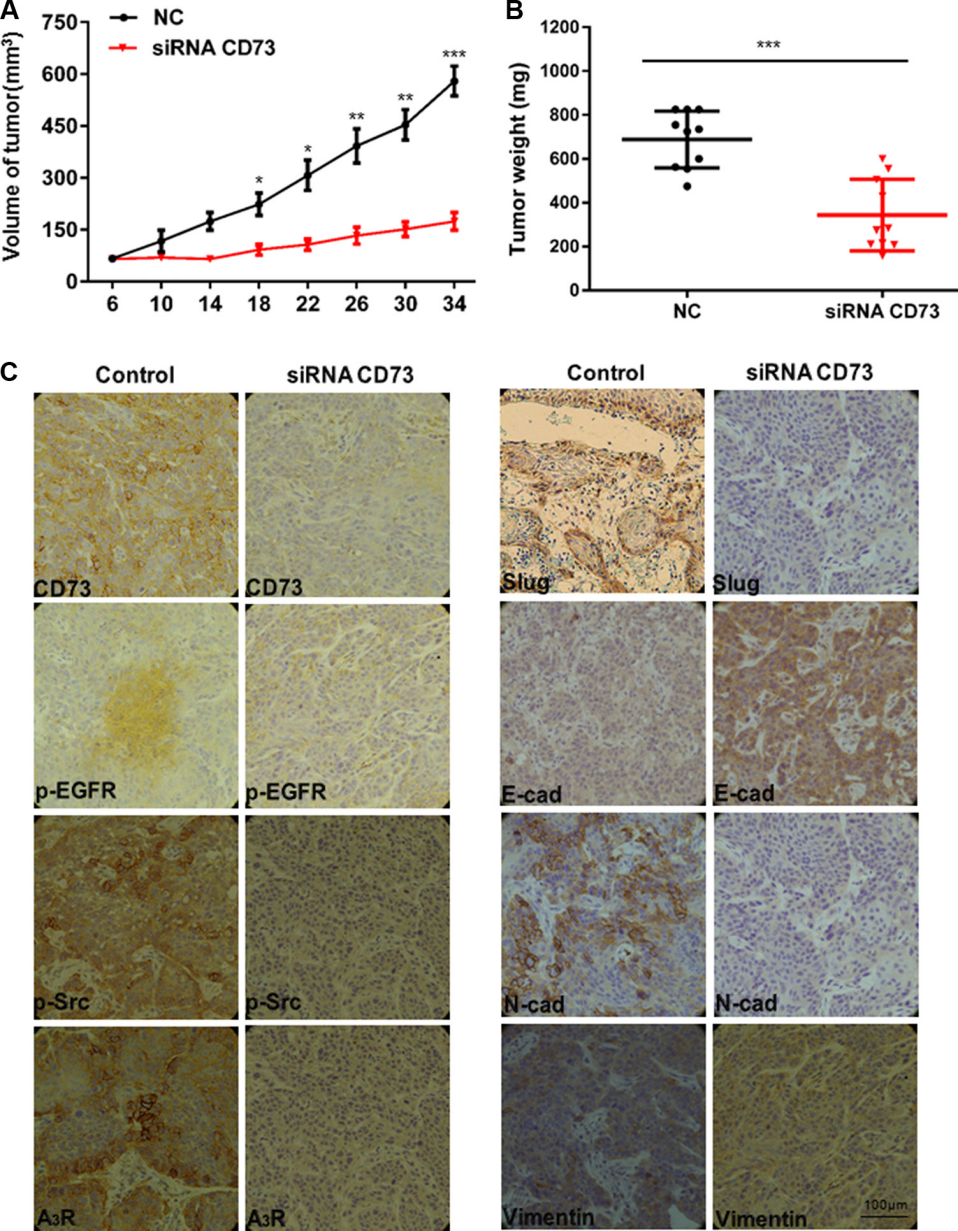
si-CD73 inhibits HNSCC tumor growth and suppress EMT *in vivo* (**A**) Tumor growth curve of siRNA-CD73 mice and control mice. Data represent the mean ± SEM. of eight mice in each group. *P < 0.05, **P < 0.01, ***P < 0.001 by the Student's *t*-test; (**B**) Dissected tumors were photographed. The tumor volume and weight were measured. ***P < 0.001 by the Student's *t*-test; (**C**) Representative images of immunohistochemical analysis of CD73, p-EGFR, p-Src, A_3_R, Slug, E-cad, N-cad and Vimentin in tumors, (*Scale bars* = 100 μm).

Based on our *in vitro* findings, we investigated whether CD73 could promote invasion and metastasis of HNSCC *in vivo*. A xenograft tumor model was established via subcutaneous injection of CAL27 and HN4 cells into nude mice. siRNA-mediated suppression of CD73 significantly inhibited tumor growth, and reduced tumor volume (*P* < 0.001) (Figure 5A) and tumor weight (*P* < 0.001) (Figure 5B). Immunohistochemistry confirmed that CD73 knockdown resulted in suppression of N-cadherin and Slug, partial suppression of Vimentin, and increased E-cadherin expression (Figure 5C). These findings suggest that knockdown of CD73 could inhibit EMT progression and that targeting CD73 can reduce tumor growth, invasion and metastasis of HNSCC cells *in vivo*.

## DISCUSSION

Previous studies have demonstrated that CD73 expression correlates with increased invasion, migration, and lymph node metastasis of breast [19], ovarian [18] and prostate cancers alike [20]. Moreover, some groups have reported that CD73 expression correlates with metastasis in breast cancer cell lines and in mouse models of this disease [21]. However, little is known about the role of CD73 gene in HNSCC. In the present study, we conducted *in vitro* and *in vivo* experiments to determine the clinical significance of CD73 in HNSCC and further characterize the molecular mechanisms by which this gene contributes to disease pathogenesis.

Our results revealed that CD73 is significantly associated with a poor prognosis in HNSCC. Both gene expression and protein expression analyses confirmed the prognostic significance of CD73 in HNSCC, thus consistent with findings reported in other cancers. Overexpression of CD73 has been observed in numerous types of cancers, and its clinical significance has also been confirmed by correlative analysis. In breast cancer, Loi *et al.* demonstrated that CD73 expression was significantly associated with a worse prognosis in triple negative breast cancer patients [15]. In cancers of the digestive system, researchers evaluated the clinical significance and prognostic value of CD73 in human gastric [16] and gallbladder cancers [17]. They revealed that the overall survival rate was low in patients with high expression of CD73. Moreover, overexpression of CD73 positively correlated with tumor differentiation, depth of invasion, nodal status, metastasis, and cancer stage. In hematologic neoplasms, Serra *et al.,* investigated the clinical significance of CD73 in chronic lymphoblastic leukemia and found that overexpression of CD73 was associated with a more aggressive clinical behavior [22].

During tumor progression and metastasis, tumor cells employ multiple pathways to evade immune surveillance [23, 24], including changes in adenosine signaling [25–27]. CD73 plays a critical role in catalyzing the hydrolysis of AMP into adenosine [28]. To mediate its immunosuppressive function, CD73 generated adenosine can bind to 4 distinct G-protein-coupled receptors: A_1_R, A_2A_R, A_2B_R, and A_3_R, exerting its effect on immune system through multiple pathways [12, 29, 30].

We found that CD73 could promote invasion and metastasis of HNSCC through adenosine receptor (especially in adenosine receptor 3) stimulation and confirmed that CD73 and adenosine signaling played a crucial role in tumor progression and metastasis. However, it should be noted that the adenosine receptor A_3_R, but not A_2A_R or A_2B_R, played the most important role in tumor progression and metastasis of HNSCC. Notably, our results conflict with previous publications. The vast majority of published research suggests that A_2A_R or A_2B_R are the functional adenosine receptors, and thus contribute to suppression of antitumor immune response and promote tumor growth [18, 31–33]. We believed that the main reason for the discrepancy between our results and those previously published findings is the specific cancer types investigated, however this hypothesis needs to be confirmed in future studies.

Another finding of our study was the observation that CD73 enhanced invasion and metastasis of HNSCC cells, mediated by activation of EGF/EGFR signaling, and its downstream phosphorylation of FAK. Some studies [34–37] have suggested CD73 promotes cancer cells proliferation via other molecules, independent of adenosine, such as EGFR. By example, Zhi *et al.* found that CD73 modulated EGFR expression and phosphorylation in human breast cancer [19]. More recently, studies have suggested that CD73 expression may serve as a potential marker for cetuximab in colorectal cancer and implicated the HER axis signaling and immune modulation as potential mechanisms of cetuximab action and sensitivity [38, 39]. While our results support the above conclusion, these results need to be confirmed in future studies.

In summary, our data demonstrates that CD73 is associated with HNSCC with poor prognosis. CD73 mediated adenosine signaling pathways and EGFR phosphorylation to improve malignant behaviors of HNSCC. Thus, CD73 may be a potential prognostic biomarker, and a therapeutic target in HNSCC. Our study thus sheds new light on the protumorigenic effects of CD73 in HNSCC.

## MATERIALS AND METHODS

### Patient population, generation of stable cell lines and reagents

We performed a retrospective analysis of atients diagnosed with HNSCC at the Shanghai Ninth People's Hospital, between December 2006 to 2008. Following central pathology review, tumors with a histology other than squamous cell carcinoma were excluded. Patients who received radiation, chemotherapy or other treatments before surgery were also excluded. 162 patients were included in the final analysis. This study was approved by the Ethics Committee of Ninth People's Hospital, Shanghai Jiao Tong University School of Medicine. Informed consent was obtained from each patient.

Human HNSCC cell lines CAL27 was obtained from the American Type Culture Collection (Manassas, VA). WSU-HN4 was obtained in December 2008 from the laboratory of Dr. Li Mao (University of Maryland Dental School, Maryland, USA). Both cell lines were maintained in Dulbecco's modified Eagle's medium (DMEM) and the HMS-001 in DMEM/F12 supplemented with 10% FBS, L-glutamine, sodium pyruvate, nonessential amino acids, and a vitamin solution, and incubated at 37°C in 5% CO_2_ and 95% Air. The identity of both cell lines was authenticated using short tandem repeat testing within 6 months of cell use. CD73 gene silencing was performed using lentiviral vectors expressing a siRNA-encoding plasmid targeting human CD73 (NM-002526; Neuron Biotech) or GFP as control (target sequence: 50-GCCGCTTTAGAGAATGCAACA-30) followed by a one-week selection in 1 mg/mL puromycin. Stable silencing of CD73 was assessed by western blot.

Sodium stibogluconate (SSG, a Src activator), PP2 (Src kinase inhibitor), adenosine receptor 1 (A_1_R) 2-chloro-N (6)-cyclopentyladenosine (CCPA), adenosine receptor 2A (A_2A_R) ALT-146e, adenosine receptor 2B (A_2B_R) BAY-60-6583, and adenosine receptor 3 (A_3_R) MRS3588 were purchased from Sigma-Aldrich. Antibodies against GAPDH were purchased Santa Cruz Biotechnology (Santa Cruz, CA) while E-cadherin, N-cadherin, vimentin, Twist, Snail and Slug were purchased from Cell Signaling Technology Inc. (Beverley, MA).

### Wound healing assay

CAL27 or WSU-HN4 cells were seeded in 6-well plates (Corning Life Sciences, USA) at 1.0 × 10^5^ cells/well. When cells reached 80% confluence, the center of each well was scratched with a sterile pipette tip to generate a constant gap, and the cells were allowed to incubate with DMEM medium without FBS for an additional 36 hours. After fixation, cells were photographed under phase microscopy and counted as previously described.

### Transwell invasion assays

Transwell (6.5 mm) with 8 μm pore polycarbonate membrane inserts (Corning, Albany, NY) were embedded with 120 μg matrigel (BD Biosciences, San Jose, CA, USA) and 100 μg gelatin (Sigma-Aldrich, St Louis, MO, USA) in DMEM. CAL27 or WSU-HN4 cells (1 × 10^5^ per well) were added to the Matrigel-embedded inserts (the top chambers) in serum-free medium, and the inserts were placed into the bottom chambers containing 10% FBS media. The cells in the upper chamber were carefully removed with cotton swab after incubation for 36 h at 37°C. Cells that had invaded through Matrigel were stained with Hematoxylin, photographed and quantified.

### Quantitative real-time RT-PCR

Quantitative Real-Time RT-PCR was performed to evaluate the expression of CD73 and EGFR-related signaling genes in HNSCC cell lines. In brief, total RNA was extracted using Trizol (Takara, Kyoto, Japan) and treated with RNase-free DNase I (Takara) to avoid genomic DNA contamination. RNA aliquots (1 μg) were reverse transcribed to cDNA (20 μl) using PrimeScriptTM RT Kit (Takara). PCR amplification using 2 μl cDNA was carried out using SYBR^®^ Premix EX TaqTMIIqPCR mix (Takara) on ABI7500 Real-Time PCR system (Applied Biosystems). GAPDH or 18sRNA was used as an internal control, and reactions were run in triplicate, with the results being averaged. Table S1 showed the primer sequences used in this study. The relative expression of genes was calculated using the ΔΔCt method.

### Western blot analysis

Whole cell extracts were prepared and Western blot analysis was conducted with indicated antibodies as previously described [40, 41]. Briefly, cells were lyzed and supernatants collected. Forty micrograms of each sample were separated by SDS-PAGE to detect CD73, EGFR/p-EGFR, FAK/P-FAK, AKT/p-AKT, ERK/p-ERK, p-Src, A_1_R, A_2A_R, A_2B_R, and A_3_R expression levels with GAPDH as a protein loading control. The samples were transferred onto polyvinylidenefluoride membranes (Millipore, Billerica, MA), blocked with 5% nonfat dry milk and powder in 0.05% Tris-buffered saline and Tween 20 (TBST) for 1 h at room temperature, then incubated overnight at 4°C with specific antibodies. The membranes were washed three times and incubated for 1 h at room temperature with peroxidase-conjugated secondary antibodies. Blots were then developed by West Pico enhanced chemiluminescence detection kit (West Pico, Thermo). Primary antibodies against CD73 (IE9), EGFR (A-10), p-EGFR (Thr1068), and p-Src (9A6) were purchased from Santa Cruz Biotechnology, FAK/P-FAK (Tyr925), AKT (62A8)/p-AKT (Thr308), ERK/p-ERK (Thr202/Tyr204) were purchased from Cell Signaling Technology, A_1_R, A_2A_R, A_2B_R, and A_3_R were purchased from Novus Biologicals.

### Immunohistochemistry

Immunohistochemistry analysis was conducted with indicated antibodies as previously described [42]. Paraffin-embedded 3 μm-thick sections were deparaffinized, rehydrated and heated with citric acid buffer at 95°C for 20 min for antigen retrieval. Sections were cooled and immersed in 0.3% hydrogen peroxide for 20 min to block endogenous peroxidase activity, rinsed in phosphate-buffered saline (PBS) for 5 min and blocked with 3% bovine serum albumin (BSA) at room temperature for 20 min. Tissues were incubated with the indicated primary antibodies in a humidified chamber overnight at 4°C. After several washes with PBS, the sections were incubated with horseradish peroxidase (HRP)-labeled goat anti-mouse or goat ant-rabbit secondary antibody (Gene Tech; Shanghai, China) for 45 min at 37°C. Diaminobenzene was used as the chromogen, and hematoxylin was used to counterstain nuclei. The sections were dehydrated, cleared and mounted. Staining was independently evaluated by an expert pathologist, who was blinded to the clinical information.

The correlation between CD73 and adenosine receptors was investigated by double immunostaining of CD73, using 4 adenosine receptors. CAL27 or HN4 cells were seeded onto coverslips at a density of 10^5^/mL and cultured in a 6-well plate for 24 hours. Cells were washed twice in PBS and fixed in 4% paraformaldehyde for half an hour. Cells were permeabilized in 0.2% Triton X-100 in PBS for 10 minutes, and blocked by non-immune goat serum for an hour at room temperature. Cells were then incubated at 4°C overnight respectively, with CD73 (1:200 dilution, Santa Cruz) and after the PBS washout, A_1_R, A_2A_R, A_2B_R, and A_3_R -conjugated secondary antibodies (1:200 dilution, Novus) were used for detection and DAPI for nucleus counterstaining. The coverlips were mounted on microscope slides with antifade mounting media (Molecular Probes, Carlsbad, CA, USA) and photographed with a fluorescence microscope (Leica).

### Animal studies

All animal proposals were approved and supervised by the institutional animal care and use committee of Ninth People's Hospital, Shanghai Jiao Tong University School of Medicine. Institutional guidelines for the proper and humane use of animals in research were followed. Male BALB/c nude mice 4–6 weeks of age were housed in the Experimental Animal Center of Ninth People's Hospital, Shanghai Jiao Tong University School of Medicine in pressurized ventilated cages according to institutional regulations. CAL27 cells (1 × 10^6^ in 0.1 mL of medium) (or HN4 cells, 2 × 10^6^ in 0.1 mL of medium) were inoculated subcutaneously into the flank of mice as control group (*n* = 4). CAL27 CD73 siRNA cells (1 × 10^6^ in 0.1 mL of medium) (or HN4 cells, 2 × 10^6^ in 0.1 mL of medium) were inoculated subcutaneously into the flank of these mice (*n* = 4) in the experimental group. After 6 weeks, the mice were sacrificed, and tumor weight was recorded.

### Statistical analysis

All data is presented as mean ± SEM. Data were analyzed and visualized using Graph-Pad Prism 5.0. One way analysis of variance followed by post Tukey Test was used to determine statistical differences between control group and treatment group. Kaplan-Meier survival curves for different strata were plotted for overall survival and disease-free survival. All experiments were independently repeated in triplicate. All tests were two-sided and no corrections were applied for multiple significance testing, with significance was defined as a *p* < 0.05, Statistical analysis was conducted using IBM SPSS Statistics 20 (IBM Corporation, NY, USA).

## SUPPLEMENTARY MATERIALS FIGURES AND TABLE



## References

[1] Zhi X, Lamperska K, Golusinski P, Schork NJ, Luczewski L, Kolenda T, Golusinski W, Masternak MM (2015). Gene expression analysis of head and neck squamous cell carcinoma survival and recurrence. Oncotarget.

[2] Song Y, Li L, Ou Y, Gao Z, Li E, Li X, Zhang W, Wang J, Xu L, Zhou Y, Ma X, Liu L, Zhao Z (2014). Identification of genomic alterations in oesophageal squamous cell cancer. Nature.

[3] Jemal A, Bray F, Center MM, Ferlay J, Ward E, Forman D (2011). Global cancer statistics. CA Cancer J Clin.

[4] Quan J, Johnson NW, Zhou G, Parsons PG, Boyle GM, Gao J (2012). Potential molecular targets for inhibiting bone invasion by oral squamous cell carcinoma: a review of mechanisms. Cancer Metastasis Rev.

[5] Ren ZH, Wu HJ, Zhang S, Wang K, Gong ZJ, He ZJ, Peng J (2015). A new surgical strategy for treatment of tongue squamous cell carcinoma based on anatomic study with preliminary clinical evaluation. J Craniomaxillofac Surg.

[6] Ren ZH, Xu JL, Fan TF, Ji T, Wu HJ, Zhang CP (2015). The Harmonic Scalpel versus Conventional Hemostasis for Neck Dissection: A Meta-Analysis of the Randomized Controlled Trials. PloS one.

[7] Ren ZH, Xu JL, Li B, Fan TF, Ji T, Zhang CP (2015). Elective versus therapeutic neck dissection in node-negative oral cancer: Evidence from five randomized controlled trials. Oral Oncol.

[8] Li H, Zhang J, Chen SW, Liu LL, Li L, Gao F, Zhuang SM, Wang LP, Li Y, Song M (2015). Cancer-associated fibroblasts provide a suitable microenvironment for tumor development and progression in oral tongue squamous cancer. J Transl Med.

[9] Ren ZH, Zhang CP, Ji T (2016). Expression of SOX2 in oral squamous cell carcinoma and the association with lymph node metastasis. Oncol Lett.

[10] Ren ZH, Wu HJ, Ji T, Wang K, Gokavarapu S, Zhang CP (2016). Clinical Application of an Original Vascular Anastomosis: A Clinical Multicenter Study. J Oral Maxillofac Surg.

[11] Ghiringhelli F, Bruchard M, Chalmin F, Rebe C (2012). Production of adenosine by ectonucleotidases: a key factor in tumor immunoescape. J Biomed Biotechnol.

[12] Allard B, Pommey S, Smyth MJ, Stagg J (2013). Targeting CD73 enhances the antitumor activity of anti-PD-1 and anti-CTLA-4 mAbs. Clin Cancer Res.

[13] Zhi X, Chen S, Zhou P, Shao Z, Wang L, Ou Z, Yin L (2007). RNA interference of ecto-5′-nucleotidase (CD73) inhibits human breast cancer cell growth and invasion. Clin Exp Metastasis.

[14] Terp MG, Olesen KA, Arnspang EC, Lund RR, Lagerholm BC, Ditzel HJ, Leth-Larsen R (2013). Anti-human CD73 monoclonal antibody inhibits metastasis formation in human breast cancer by inducing clustering and internalization of CD73 expressed on the surface of cancer cells. J Immunol.

[15] Loi S, Pommey S, Haibe-Kains B, Beavis PA, Darcy PK, Smyth MJ, Stagg J (2013). CD73 promotes anthracycline resistance and poor prognosis in triple negative breast cancer. Proc Natl Acad Sci USA.

[16] Lu XX, Chen YT, Feng B, Mao XB, Yu B, Chu XY (2013). Expression and clinical significance of CD73 and hypoxia-inducible factor-1alpha in gastric carcinoma. World J Gastroenterol.

[17] Xiong L, Wen Y, Miao X, Yang Z (2014). NT5E and FcGBP as key regulators of TGF-1-induced epithelial-mesenchymal transition (EMT) are associated with tumor progression and survival of patients with gallbladder cancer. Cell Tissue Res.

[18] Turcotte M, Spring K, Pommey S, Chouinard G, Cousineau I, George J, Chen GM, Gendoo DM, Haibe-Kains B, Karn T, Rahimi K, Le Page C, Provencher D (2015). CD73 Is Associated with Poor Prognosis in High-Grade Serous Ovarian Cancer. Cancer Res.

[19] Zhi X, Wang Y, Yu J, Yu J, Zhang L, Yin L, Zhou P (2012). Potential prognostic biomarker CD73 regulates epidermal growth factor receptor expression in human breast cancer. IUBMB life.

[20] Leclerc BG, Charlebois R, Chouinard G, Allard B, Pommey S, Saad F, Stagg J (2015). CD73 Expression Is an Independent Prognostic Factor in Prostate Cancer. Clin Cancer Res.

[21] Stagg J, Divisekera U, Duret H, Sparwasser T, Teng MW, Darcy PK, Smyth MJ (2011). CD73-deficient mice have increased antitumor immunity and are resistant to experimental metastasis. Cancer Res.

[22] Serra S, Horenstein AL, Vaisitti T, Brusa D, Rossi D, Laurenti L, D'Arena G, Coscia M, Tripodo C, Inghirami G, Robson SC, Gaidano G, Malavasi F (2011). CD73-generated extracellular adenosine in chronic lymphocytic leukemia creates local conditions counteracting drug-induced cell death. Blood.

[23] Hanahan D, Weinberg RA (2011). Hallmarks of cancer: the next generation. Cell.

[24] Schonhuber N, Seidler B, Schuck K, Veltkamp C, Schachtler C, Zukowska M, Eser S, Feyerabend TB, Paul MC, Eser P, Klein S, Lowy AM, Banerjee R (2014). A next-generation dual-recombinase system for time- and host-specific targeting of pancreatic cancer. Nat Med.

[25] Antonioli L, Pacher P, Vizi ES, Hasko G (2013). CD39 and CD73 in immunity and inflammation. Trends Mol Med.

[26] Bastid J, Regairaz A, Bonnefoy N, Dejou C, Giustiniani J, Laheurte C, Cochaud S, Laprevotte E, Funck-Brentano E, Hemon P, Gros L, Bec N, Larroque C (2015). Inhibition of CD39 enzymatic function at the surface of tumor cells alleviates their immunosuppressive activity. Cancer Immunol Res.

[27] Bono MR, Fernandez D, Flores-Santibanez F, Rosemblatt M, Sauma D (2015). CD73 and CD39 ectonucleotidases in T cell differentiation: Beyond immunosuppression. FEBS Lett.

[28] Young A, Mittal D, Stagg J, Smyth MJ (2014). Targeting cancer-derived adenosine: new therapeutic approaches. Cancer Discov.

[29] Sorrentino R, Pinto A, Morello S (2013). The adenosinergic system in cancer: Key therapeutic target. Oncoimmunology.

[30] Muller-Haegele S, Muller L, Whiteside TL (2014). Immunoregulatory activity of adenosine and its role in human cancer progression. Expert Rev Clin Immunol.

[31] Kumar V (2013). Adenosine as an endogenous immunoregulator in cancer pathogenesis: where to go?. Purinergic Signal.

[32] Novitskiy SV, Ryzhov S, Zaynagetdinov R, Goldstein AE, Huang Y, Tikhomirov OY, Blackburn MR, Biaggioni I, Carbone DP, Feoktistov I, Dikov MM (2008). Adenosine receptors in regulation of dendritic cell differentiation and function. Blood.

[33] Sciaraffia E, Riccomi A, Lindstedt R, Gesa V, Cirelli E, Patrizio M, De Magistris MT, Vendetti S (2014). Human monocytes respond to extracellular cAMP through A2A and A2B adenosine receptors. J Leukoc Biol.

[34] Shirali S, Aghaei M, Shabani M, Fathi M, Sohrabi M, Moeinifard M (2013). Adenosine induces cell cycle arrest and apoptosis via cyclinD1/Cdk4 and Bcl-2/Bax pathways in human ovarian cancer cell line OVCAR-3. Tumour Biol.

[35] Tsuchiya A, Nishizaki T (2015). Anticancer effect of adenosine on gastric cancer via diverse signaling pathways. World J Gastroenterol.

[36] Mello Pde A, Filippi-Chiela EC, Nascimento J, Beckenkamp A, Santana DB, Kipper F, Casali EA, Nejar Bruno A, Paccez JD, Zerbini LF, Wink MR, Lenz G, Buffon A (2014). Adenosine uptake is the major effector of extracellular ATP toxicity in human cervical cancer cells. Mol Biol Cell.

[37] Postow MA, Callahan MK, Wolchok JD (2015). Immune Checkpoint Blockade in Cancer Therapy. J Clin Oncol.

[38] Cushman SM, Jiang C, Hatch AJ, Shterev I, Sibley AB, Niedzwiecki D, Venook AP, Owzar K, Hurwitz HI, Nixon AB (2015). Gene expression markers of efficacy and resistance to cetuximab treatment in metastatic colorectal cancer: results from CALGB 80203 (Alliance). Clin Cancer Res.

[39] Kawazoe A, Shitara K, Fukuoka S, Kuboki Y, Bando H, Okamoto W, Kojima T, Fuse N, Yamanaka T, Doi T, Ohtsu A, Yoshino T (2015). A retrospective observational study of clinicopathological features of KRAS, NRAS, BRAF and PIK3CA mutations in Japanese patients with metastatic colorectal cancer. BMC cancer.

[40] Tanaka N, Patel AA, Wang J, Frederick MJ, Kalu NN, Zhao M, Fitzgerald AL, Xie TX, Silver NL, Caulin C, Zhou G, Skinner HD, Johnson FM (2015). Wee-1 Kinase Inhibition Sensitizes High-Risk HPV+ HNSCC to Apoptosis Accompanied by Downregulation of MCl-1 and XIAP Antiapoptotic Proteins. Clin Cancer Res.

[41] Shao N, Lu Z, Zhang Y, Wang M, Li W, Hu Z, Wang S, Lin Y (2015). Interleukin-8 upregulates integrin beta3 expression and promotes estrogen receptor-negative breast cancer cell invasion by activating the PI3K/Akt/NF-kappaB pathway. Cancer Lett.

[42] Ren ZH, Yuan YX, Ji T, Zhang CP (2016). CD73 as a novel marker for poor prognosis of oral squamous cell carcinoma. Oncol Lett.

