# Supplementary material for: CD73 is associated with poor prognosis in HNSCC
Source: Oncotarget. 2016 Aug 20;7(38):61690–702. doi: 10.18632/oncotarget.11435 (PMC5308683; doi:10.18632/oncotarget.11435)
Supplement: Supplementary file 1 [file oncotarget-07-61690-s001.pdf]

CD73 is associated with poor prognosis in HNSCC

Supplementary Materials

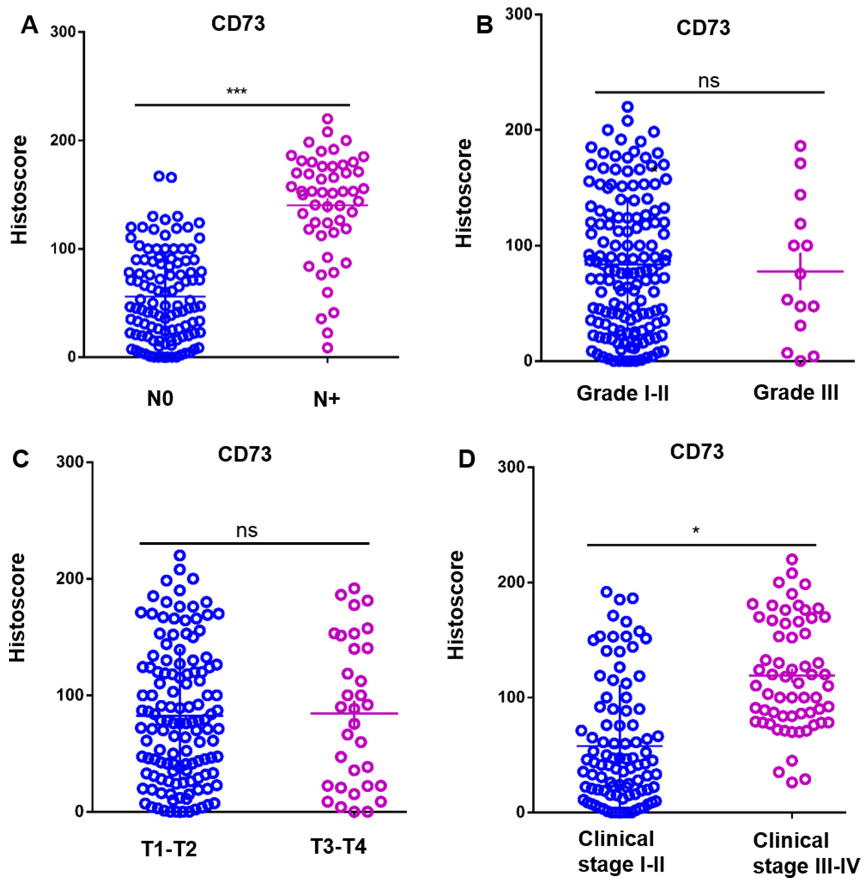

Supplementary Figure S1: CD73 expression was correlated with lymph node metastasis and clinical stages in HNSCC. (**A**) CD73 expression correlates with lymph node metastasis in HNSCC; (**B**) CD73 expression was not specifically associated with different pathological grades of HNSCC; (**C**) CD73 expression did not correlate with T category of HNSCC; (**D**) CD73 expression correlated with clinical stages of HNSCC (Mean ± SEM; *$P < 0.05$, ***$P < 0.001$, *t*-test).

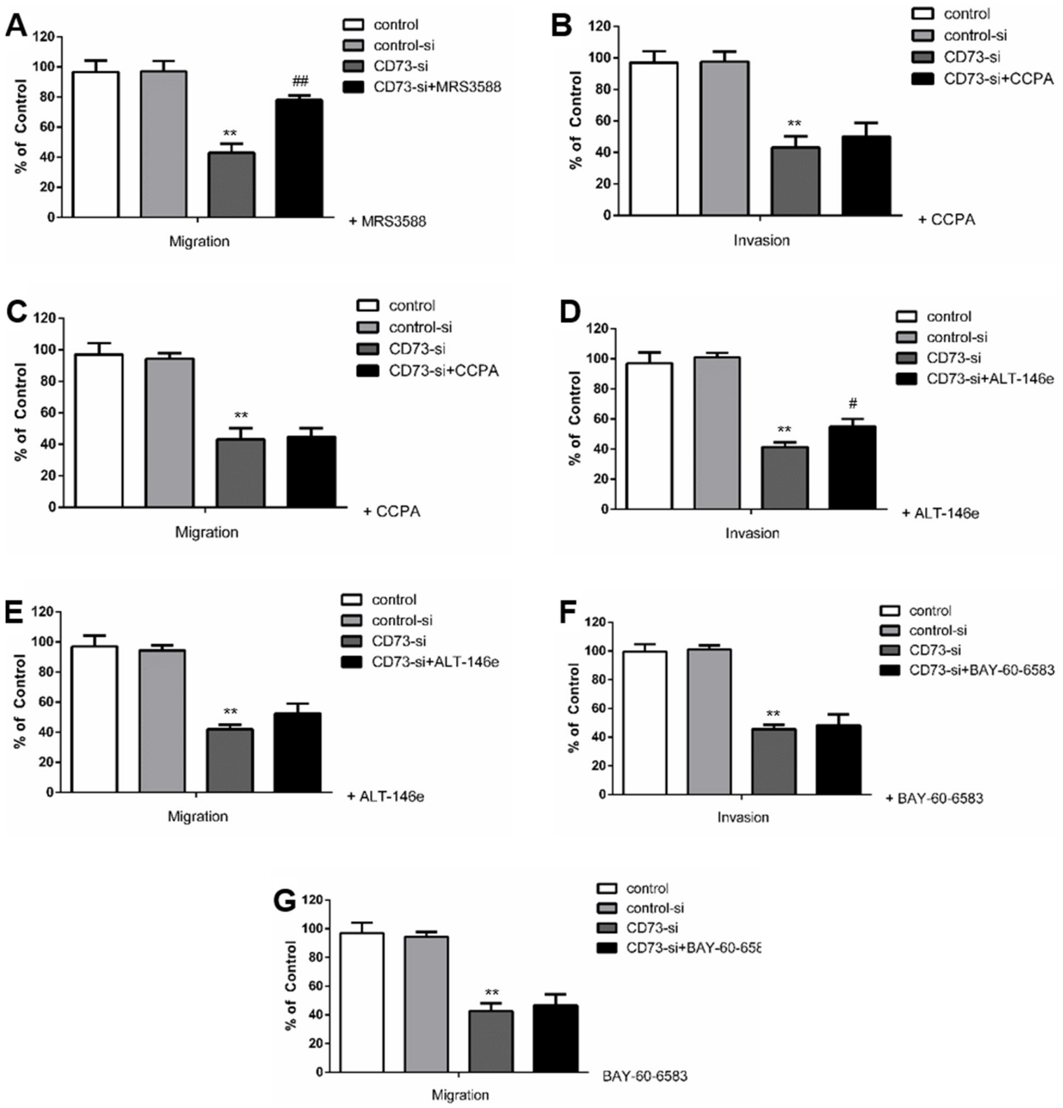

Supplementary Figure S2: The migration abilities of CAL27 would reversal after using MRS3588. (**A**) Cell migration increased significantly following treatment with MRS3588, specific A_3Ragonist; (**B**–**C**) Cell migration and invasion did not change following CCPA (2-chloro-N (6)-cyclopentyladenosine), specific A_1R agonist, treatment; (**D**) Cell invasion increased significantly following ALT-146e, specific A_{2A}R agonist treatment; (**E**) Cell migration did not change when adding ALT-146e, specific A_{2A}R agonist; (**F**–**G**) Cell migration and invasion did not change when adding BAY-60-6583, specific A_{2B}Ragonist. Quantification of cell numbers with Image J "cell counter" module (Mean ± SEM; $^{**}P < 0.01$, versus control-si group, $^{\#}P < 0.05$, $^{\#\#}P < 0.01$, versus the CD73-si group, student *t*-test with GraphPad Prism5.0).

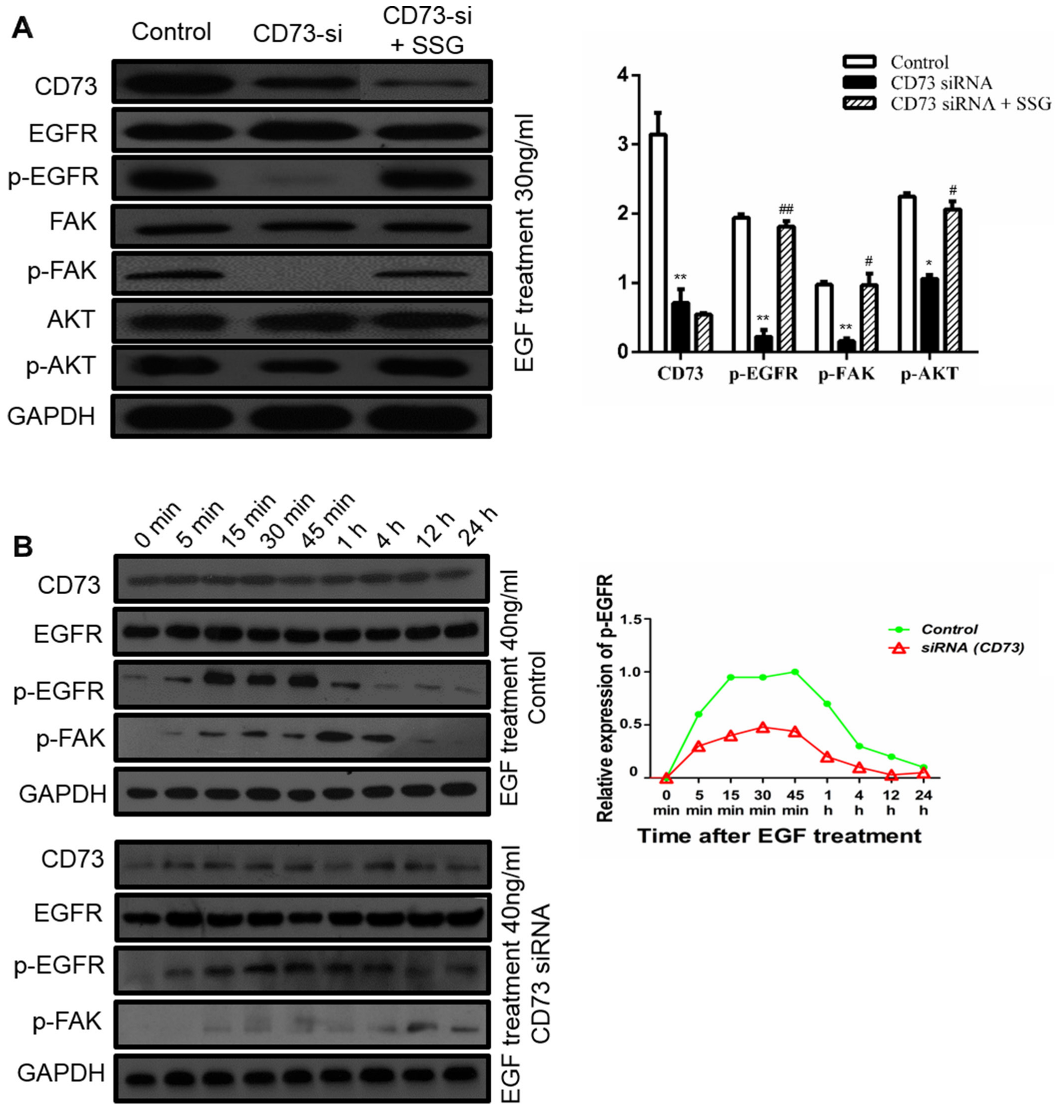

Supplementary Figure S3: CD73 activated EGFR/ FAK/ AKT pathways. (**A**) p-EGFR, p-FAK, p-AKT levels were determined following siRNA-suppression of CD73 in CAL27 cells. GAPDH was the internal standard for protein loading. The values are presented as the means ± SEM. One-way ANOVA with post-Dunnett analysis was performed using GraphPad Prism5. **P* < 0.05, ***P* < 0.01, versus the control group, $^{\#}P < 0.05$, $^{\#\#}P < 0.011$, versus the CD73-si group, (*n* = 3); (**B**) Western blot examined the time-dependent phosphorylation of EGFR and PAK after EGF treatment in both CAL27 control-si and CAL27 CD73-si groups, and graphed by Quantitative analysis.

Supplementary Table S1: Primer sequence

Gene	Primer	Sequence (5′ to 3′)
CD73	Forward	GCCTGGGAGCTTACGATTTG
	Reverse	TAGTGCCCTGGTACTGGTCG
GAPDH	Forward	AGAAGGCTGGGGCTCATTTG
	Reverse	AGGGGCCATCCACAGTCTTC
